# Perineal Distensibility Using *Epi-no* in Twin Pregnancies: Comparative Study with Singleton Pregnancies

**DOI:** 10.1155/2014/124206

**Published:** 2014-03-27

**Authors:** Juliana Sayuri Kubotani, Antonio Fernandes Moron, Edward Araujo Júnior, Miriam Raquel Diniz Zanetti, Vanessa Cardoso Marques Soares, Julio Elito Júnior

**Affiliations:** Pelvic Floor Sector, Department of Obstetrics, Federal University of São Paulo (UNIFESP), Rua Carlos Weber, 956 Apartment 113 Visage, Vila Leopoldina, 05303-000 São Paulo, SP, Brazil

## Abstract

The aims of this study were to compare perineal distensibility between women with twin and singleton pregnancies and to correlate these women's perineal distensibility with anthropometric data. This prospective cross-sectional case-control study was conducted among nulliparous women, of whom 20 were pregnant with twins and 23 with a single fetus. Perineal distensibility was evaluated in the third trimester by means of *Epi-no*, which was introduced into the vagina and inflated up to the maximum tolerable limit. It was then withdrawn while inflated and its circumference was measured. The unpaired Student's *t*-test was used to compare perineal distensibility in the two groups and Pearson's correlation coefficient (*r*) was used to correlate the pregnant women's perineal distensibility with their anthropometric data. There was no difference in perineal distensibility between the twin group (16.51 ± 2.05 cm) and singleton group (16.13 ± 1.67 cm) (*P* = 0.50). There was a positive correlation between perineal distensibility and abdominal circumference (*r* = 0.36; *P* = 0.01). The greater the abdominal circumference was, the greater the perineal distensibility was, regardless of whether the pregnancy was twin or singleton.

## 1. Introduction

The term pelvic floor refers to all of the muscles, connective tissue, and organs that fill the cavity of the pelvic canal. The muscles of the pelvic floor form a diaphragm that encompasses the pelvic cavity. Their fibers have a U shape around the hiatus, which allows this to be constantly closed, thus providing pelvic support for the abdominal organs [1].

During pregnancy, with uterine growth, the pelvic floor becomes overloaded and, because of the influence of hormones and biomechanical changes to the pelvis, its tonus and strength diminish [2], and urinary symptoms can be observed even before delivery [3].

The* Epi-no* Delphine Plusvaginal dilator (Starnberg Medical, Tecsana GmbH, Munich, Germany) consists of an inflatable silicone balloon connected to a manometer via a rubber tube [4, 5]. In the absence of any instrument that could quantify this stretching, the* Epi-no* apparatus was adapted to objectively and quantitatively evaluate the degree of perineal distensibility.

In twin pregnancies, the gestational changes are more pronounced, given that there are two fetuses, two placentas, and amniotic fluid for two fetuses, thus producing an even greater overload on the pelvic floor. From this supposition, the purposes of the present study were to compare the degree of distensibility of the musculature of the pelvic floor among women with twin pregnancies with that of women with singleton pregnancies, by means of the* Epi-no* vaginal dilator, and to correlate these pregnant women's perineal distensibility with their anthropometric data.

## 2. Materials and Methods

A prospective cross-sectional case-control study was conducted between August 2011 and April 2013. For this, 20 women with twin pregnancies and 23 with singleton pregnancies aged between 20 and 38 years, all nulliparous, were selected. The pregnant women came from the outpatient clinics of the Department of Obstetrics, Federal University of São Paulo (UNIFESP), at gestational ages of between 20 and 38 weeks. This study was approved by the Research Ethics Committee of UNIFESP under the number 0506/11, and all the pregnant women signed a free and informed consent statement.

Pregnant women who presented the following dysfunctions of the pelvic floor were excluded: urinary or fecal incontinence or genital prolapse prior to pregnancy; fetal abnormalities detected in ultrasonography examinations; previous fetal death at a gestational age of more than 20 weeks; monochorionic twin pregnancies complicated by twin-to-twin transfusion syndrome, acardiac fetus or conjoined twins; or multiple pregnancies with three or more fetuses.

To evaluate perineal distensibility, the pregnant women were positioned in dorsal decubitus with their lower limbs flexed and abducted at between 30° and 45° and with their feet supported on the bed. The woman was instructed not to perform contraction of the perineal, gluteal, or adductor musculature or to perform a Valsalva maneuver. The* Epi-no* balloon was introduced into the vagina in a deflated condition, enclosed in a gel-lubricated condom, to a depth that would allow 2.0 cm of the balloon to be viewed outside of the vagina [6]. In this way, no risk would be presented to the pregnant woman because the device would not reach the uterine neck. The* Epi-no *balloon was then gradually inflated until the pregnant woman signaled that she perceived or felt that the distension had reached its maximum tolerable limit. The balloon was then withdrawn without deflating it, delicately, such that the woman would not apply force to resist or assist the withdrawal, and the circumference of the balloon was measured using a measuring tape.

To determine the number of subjects needed to this study, we used Cochran's formula *n* = *t* ^2^ *xPx*(1 − *P*)/*d* ^2^ [7]. Considering a normal distribution to “*P*,” we can adopt *t* = 1.96, which means that the area under the normal curve will have size 0.05; furthermore, we will adopt a margin of error of 5.0%. “*P*” was fixed in 1% (0.01) and therefore (1 − *P*) has value of 0.99. Thus, with a margin of error of 5.0%, we would need minimum of 16 subjects to each group.

The data were transferred to a spreadsheet in the Excel 2007 software (Microsoft Corp., Redmond, WA, USA) and were analyzed using the Statistical Package for the Social Sciences (SPSS) software for Windows, version 15.0 (SPSS Inc., Chicago, IL, USA). To compare the data obtained from analysis on the* Epi-no* circumference measurements, between the two study groups, the unpaired Student's *t*-test was used. To correlate perineal distensibility with the pregnant women's anthropometric data, Pearson's correlation coefficient (*r*) was used. The significance level of *P* < 0.05 was used in all of the analyses.

## 3. Results

The mean maternal age in the group of women with singleton pregnancies (*n* = 23) was 29.82 ± 6.10 years, while in the group with twin pregnancies (*n* = 20) it was 26 ± 4.35 years (*P* = 0.03). The mean gestational age at the time of the evaluation in the group with singleton pregnancies was 32.68 ± 2.30 weeks, while in the group with twin pregnancies, it was 31.77 ± 1.42 weeks (*P* = 0.12). The mean uterine height and abdominal circumference were 30.36 ± 2.34 cm and 99.67 ± 6.84 cm, respectively, in the pregnant women with singletons, while among the women with twin pregnancies these were 35.15 ± 2.58 cm and 108.64 ± 7.61 cm, respectively, with *P* < 0.01. Regarding the gestational body mass index (BMI), the women with singletons had a mean of 26.31 ± 3.46 kg/cm^2^  and those with twins presented 29.51 ± 4.94 kg/cm^2^  (*P* = 0.01) (Table 1).

The mean circumference of the* Epi-no* balloon among all the pregnant women was 16.31 ± 1.85 cm. In the group with twins, it was 16.51 ± 2.05 cm, while, in the group with singletons, it was 16.13 ± 1.67 cm (*P* = 0.50) (Table 2).

**Figure 1:**
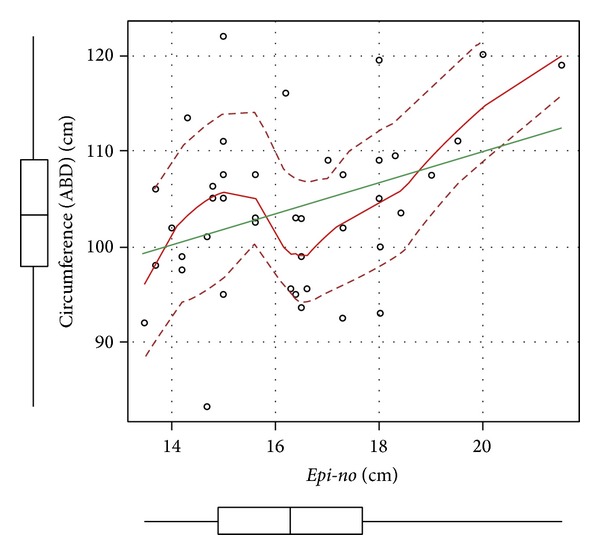
Graphical representation of the positive correlation between abdominal circumference (ABD) and measurements on the* Epi-no* balloon circumference.

There was a positive and statistically significant correlation between abdominal circumference and the circumference of the* Epi-no* balloon (*r* = 0.36; *P* = 0.01) and a trend in the correlation between BMI and* Epi-no* (Table 3 and Figure 1).

## 4. Discussion

Pregnancy causes biomechanical, neurological, and neuromuscular modifications to the pelvic floor through mechanical and hormonal effects [8]. Examples of mechanical effects include increased weight, overload generated through the fetal weight, and postural changes, while the hormonal effects come mainly from the action of progesterone and relaxin. The latter is responsible for greater joint flexibility, thus leading to the increased pelvic mobility that is evident from the start of the third gestational trimester [9, 10].

In twin pregnancies, the characteristics of both the overload on the pelvic floor and the hormonal changes are presented more intensely than in singleton pregnancies. Some authors have correlated twin pregnancies and delivery type with conditions of stress urinary incontinence, urge urinary incontinence, fecal incontinence, and gas incontinence using questionnaires alone [11–13], without any type of physical evaluation of the pelvic floor or any comparison with women with single pregnancies.

With the aim of reducing occurrences of perineal laceration and the need for expansion incisions at the time of delivery, the* Epi-no* device was developed. Its original purpose was to assist in preparing the perineum so that episiotomy might be avoided [5]. In the present study, we adapted the* Epi-no* balloon in order to evaluate the degree of perineal distensibility: to the best of knowledge, no studies on this have yet been published in the literature, in relation to either single or twin pregnancies.

There are some difficulties relating to evaluations on perineal distensibility. Among these is the patients' fear that the examination might trigger delivery labor, even after explanation that the* Epi-no* balloon does not reach the uterine neck, and despite the fact that no study in the literature has ever demonstrated that* Epi-no* would be able to stimulate delivery labor. Psychological issues, especially among women with twin pregnancies, who are always advised by the medical team regarding the risks of prematurity, may have an influence on the examination, given that this examination has a direct relationship with the patient's discomfort, through indication of the insufflation limit of the* Epi-no* balloon.

From a more detailed analysis in which we grouped the women with twin and singleton pregnancies, we observed that the greater the abdominal circumference was, the greater the circumference of the* Epi-no* balloon was. Moreover, there was a tendency for the distensibility of the pelvic floor to be greater with greater BMI, with the observation that there was greater overload on the pelvic floor through increased continual pressure on this.

## 5. Conclusion

In summary, we did not observe any significant differences between the twin and singleton pregnancy groups with regard to perineal distensibility, as assessed using the* Epi-no* balloon. On the other hand, we observed a positive correlation between perineal distensibility and the mother's abdominal circumference.

## Figures and Tables

**Table 1:** Patient distribution according to demographic and anthropometric characteristics.

Variable	Group	*N*	Mean	Standard deviation	Significance (*P*)
Age	singleton	23	29.82	6.10	0.03*
	twin	20	26	4.35	

GA	singleton	23	32.68	2.30	0.12**
	twin	20	31.77	1.42	

UH	singleton	22	30.36	2.34	<0.01**
	twin	20	35.15	2.58	

AC	singleton	22	99.67	6.84	<0.01**
	twin	19	108.64	7.61	

BMI	singleton	22	26.31	3.46	0.01**
	twin	20	29.51	4.94	

GA: gestational age; UH: uterine height; AC: abdominal circumference; BMI: body mass index.

*Mann Whitney.

**Unpaired Student's *t*-test.

**Table 2:** Comparison of data from measurements on *Epi-no* balloon circumference between the twin and singleton pregnancy groups.

*Epi-no* balloon circumference	*n*	Minimum	Maximum	Mean	Standard deviation	*P^∗^*
Twin pregnancy	20	14	21.5	16.51	2.05	0.50
Singleton pregnancy	23	13.5	19.5	16.13	1.67	

*Unpaired Student's *t*-test.

**Table 3:** Correlation between the pregnant women's anthropometric data and their measurements from the *Epi-no* balloon circumference.

Pair of variables	*r*	*P*	Interval
MA versus *Epi-no *	−0.07	0.62	[−0.36, 0.22]
GA versus *Epi-no *	0.03	0.84	[−0.27, 0.32]
UH versus *Epi-no *	0.20	0.19	[−0.10, 0.48]
BMI versus *Epi-no *	0.28	0.06	[−0.02, 0.54]
AC versus *Epi-no *	0.36	0.01*	[0.06, 0.59]

MA: maternal age; GA: gestational age; UH: uterine height; AC: abdominal circumference; BMI: body mass index; *r*: Pearson's correlation coefficient.

*Unpaired Student's *t*-test.
